# Fixed-wing air transport of patients with spinal pathologies: a scoping review of current evidence and future research priorities

**DOI:** 10.1007/s00701-026-06852-0

**Published:** 2026-04-02

**Authors:** Ashviniy Thamilmaran, Zak Hodgson, Konstantinos Peramatzis, Maria Karampouga, Mark Jarratt, Eleni Tsianaka, Insa K. Janssen, Anastasia Tasiou, Nese Keser, Mary Murphy

**Affiliations:** 1 University College London Medical School, London, UK; 2 School of Medicine, University of Leeds, Leeds, UK; 3 Department of Aviation Engineering, School of Aviation, Australian University, Safat, Kuwait; 4 Department of Neurosurgery, Nicosia General Hospital, Nicosia, Cyprus; 5 Spinal Injuries Association, Health and Care Quality Team, Milton Keynes, UK; 6 Department of Neurosurgery & Quality Department, Kuwait Hospital, Sabah Al Salem, Kuwait; 7 Department of Neurosurgery, Hôpitaux Universitaires de Genève, Geneva, Switzerland; 8 Department of Neurosurgery, University Hospital of Larissa, Larissa, Greece; 9 Department of Neurosurgery, Hamidiye Faculty of Medicine, University of Health Sciences, Istanbul, Turkey; 10 Department of Neurosurgery, National Hospital for Neurology and Neurosurgery, London, UK

**Keywords:** Fixed-wing air transport, Spinal cord injury, Aeromedical evacuation, Spinal trauma, Altitude physiology, Aerospace medicine

## Abstract

**Background:**

Rapid transportation of neurosurgical patients, particularly those with acute spinal trauma, is critical due to the direct association between total prehospital time and mortality. While existing aeromedical guidelines focus primarily on helicopter (rotor-wing) transport, there is limited evidence consolidating the effects of fixed-wing aircraft transport, which exposes patients to unique physiological stressors due to higher operational altitudes.

**Methods:**

Using a recognised framework for scoping reviews, a European-based research team conducted biweekly discussions to ensure a comprehensive approach. This scoping review utilized a four-phase framework: identification, screening, data extraction, and contextualisation. A systematic literature search via OVID databases up to September 19, 2024, and manual searches of 15 journals identified studies involving fixed-wing aircraft transport for spinal pathology patients, excluding helicopter or animal studies. Additionally, a panel of experts in neurosurgery and aviation reflected on their experiences and transferable lessons.

**Results:**

12 studies were included, covering 105 spinal injury cases and 1 spinal tuberculosis case. Key physiological factors unique to fixed-wing transport include reduced atmospheric pressure, low humidity, extended transport duration, smoother acceleration/deceleration, and better medical accessibility compared to rotor-wing transport. Recommendations from the literature and expert experiences emphasise respiratory stability, humidification, oxygen supplementation, pressure injury prevention, bowel/bladder management, airway cuff pressure management, and venous thromboembolism prophylaxis.

**Conclusion:**

There is an urgent need for evidence-based recommendations tailored to fixed-wing transportation of spinal pathology patients, emphasizing altitude-induced physiological changes to optimize patient safety. Further targeted research is essential to develop comprehensive clinical protocols.

**Supplementary Information:**

The online version contains supplementary material available at 10.1007/s00701-026-06852-0.

## Introduction

Research has shown a linear association between total prehospital time and in-hospital mortality, regardless of case complexity [23]. Neurosurgical patients, especially those with acute spinal pathologies, are vulnerable due to the interplay between intercranial pressure (ICP), haemodynamic instability, and the risk of irreversible nerve damage. Minimising prehospital time is therefore critical to prevent catastrophic deterioration, making air medical transport a vital component of emergency care.

Where rotor-wing transport is limited, fixed-wing aircraft enable rapid transfer over long distances, inaccessible terrain, or in adverse weather. Operating at altitudes up to ten times higher than rotor-wing [54], they expose patients to distinct physiological stressors. These effects are amplified in major trauma and require careful consideration, particularly modifiable factors, to minimise harm. Altitude-related changes: reduced atmospheric pressure, altered gas exchange, and vibration, can significantly impact acute spinal trauma. Understanding these relationships is essential to optimise safety.

Most available data come from modern military aeromedical evacuation cohorts [8, 30, 47] and experimental animal studies simulating flight conditions [6, 49, 56]. Civilian literature remains sparse, and existing guidelines focus predominantly on rotor-wing operations.

This scoping review addresses this gap by consolidating published evidence on fixed-wing transport for patients with acute spinal pathology. We summarise physiological considerations, risk mitigation strategies, and relevant recommendations, and supplement this synthesis with our own clinical experience to contextualise findings and guide best practice.

### Methods

We followed the methodological framework of Arksey and O’Malley (2002) [3] to conduct this scoping review. A collaborative group of neurosurgeons across Europe met fortnightly for one year via Microsoft Teams® (version 4.2.4.0). Invited experts discussed the physiological impacts of aeronautical transfer at altitudes above helicopter operational ranges for patients with spinal pathologies, ensuring relevance and comprehensiveness.

Our strategy comprised four phases:

1. Identifying papers
2. Screening for inclusion
3. Data extraction and evidence grading
4. Contextualising findings with expert input

**Fig. 1:**
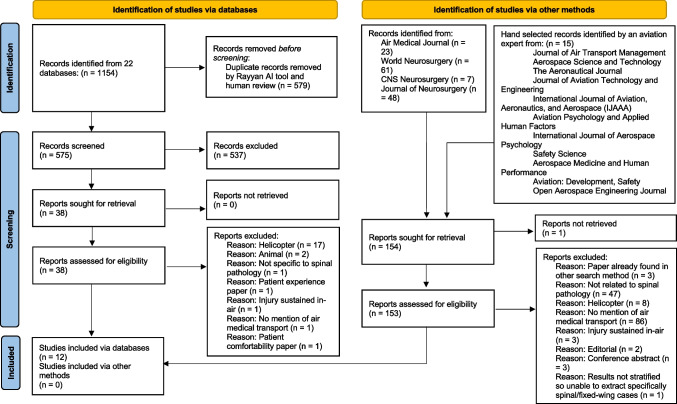
Flowchart summarising phases 1 and 2, figure adapted from PRISMA 2020 Flowchart [45]

In phase one, we performed a comprehensive OVID search across all available databases to September 19, 2024, combining subject headings and keywords for spinal pathology and aeronautical transfer. We also hand-searched the four most relevant journals (Fig. 1). Searches were limited to English-language articles and excluded animal studies. An aviation and electronics expert (KP) additionally hand-searched 11 further journals, yielding no unique studies. Detailed search strategies are in Appendix 1.

Phase two involved applying refined inclusion and exclusion criteria. We included studies on spinal pathology involving aeronautical transfer but excluded helicopter-only transfers, as these share acceleration/deceleration forces, vibration, noise, and spatial constraints but differ in atmospheric conditions, temperature, humidity, and remoteness from ground support. Australian flying doctor’s service cases with transfers ≤ 200 miles were excluded, as such flights are at lower altitudes than typical fixed-wing operations.

Screening occurred in two stages: title/abstract review and full-text assessment. Two independent reviewers (AT, ZH) evaluated each article, resolving disagreements by discussion or with a third reviewer (MM). Twelve papers met criteria (Fig. 1).

In phase three, the team collectively reviewed each included study, extracting details relevant to fixed-wing transport of acute spinal patients. Lessons and recommendations were summarised. As most evidence comprised descriptive case reports lacking control groups, formal risk of bias assessment was not performed. Each study was graded according to the Oxford Centre for Evidence-Based Medicine framework (Table 1).

**Table 1:** Level of evidence according to the study type and the grade of recommendation based on the evidence provided in the study

Level of evidence according to study type	
Level	Type of study
1a	Systematic review (with homogeneity) of randomized controlled trials
1b	Individual randomized controlled trials (with narrow confidence intervals)
2a	Systematic review (with homogeneity) of cohort studies
2b	Individual cohort study (including low-quality randomized controlled trials)
2c	Outcomes research
3a	Systematic review (with homogeneity) of case–control studies
3b	Individual case–control study
4	Case-series (and poor-quality cohort and case–control studies)
5	Expert opinion without explicit critical appraisal, or based on physiology, bench research or “first principles”
Grade of recommendation based on the evidence provided	
Grade	Description
A	Consistent level 1 studies
B	Consistent level 2 or 3 studies OR extrapolations from level 1 studies
C	Level 4 studies OR extrapolations from level 2 or 3 studies
D	Level 5 evidence OR troubling inconsistent or inconclusive studies of any level

Figure taken from OCEBM levels of evidence working group* from “The Oxford Levels of Evidence 2” [44]

In the final phase, findings were synthesised into thematic categories and summarised narratively. Expert experience in neurosurgery and aviation was integrated to contextualise the literature and highlight trends, identify knowledge gaps, and future research priorities. Additional practical lessons from years of clinical experience were also included.

## Results

The 12 papers, published from 1989–2021, which contained information about patients with spinal conditions flown at altitude included 105 cases of spinal injury, and 1 case of spinal TB are shown in Table 2. Many cases are derived from military experiences globally. The grade of recommendation based on the evidence provided is C, as the included studies were primarily level 4 evidence.

**Table 2:** Summary of included studies and their characteristics

Title	Reference	Level	Justification
Patterns and predictors of firearm-related spinal cord injuries in adult trauma patients	Mahmassani D. et al., 2021 [35]	2b	Retrospective cohort study
A 41-year-old man with an incomplete spinal cord injury	McLean M.M. et al., 2014 [39]	5	Case study
Transportation of patients with acute traumatic cervical spine injuries	Theodore N. et al., 2013 [58]	5	Expert opinion, narrative review
Spine immobilization: prehospitalization to final destination	Kang D.G. and Lehman Jr. R.A., 2011 [30]	5	Expert opinion, narrative review
Critical care air transport team (CCATT) short term outcomes of casualties with spinal fractures moved with the vacuum spine board between 2009 and 2010	Lairet J.R. et al., 2011 [32]	4	Retrospective observational study
Medical evacuation from Vietnam of an elderly with tuberculosis spondylitis	Shieh Y.-H. and Chang J.-C., 2007 [52]	5	Case study
Management of unstable cervical spine injuries in southern Iraq during OP TELIC	Bird et al., 2005 [8]	4	Case series
A geospatial examination of specialist care accessibility and impact on health outcomes for patients with acute traumatic spinal cord injury in New South Wales, Australia: a population record linkage study	Sharwood L.N. et al., 2021 [51]	2b	Retrospective population-based cohort study
Clinical features, patterns of referral and out of hospital transport events for patients with suspected isolated spinal injury	Flabouris, A, 2001 [21]	4	Descriptive observational study/case series
Respiratory problems of air travel in patients with spinal cord injuries	Armitage J.M. et al., 1990 [4]	4	Case series
Use of traction in cervical spine fractures during interhospital transfer by aircraft	Neville S. et al., 1990 [43]	4	Case series
Stabilization of spinal injury for early transfer	Burney R.E. et al., 1989 [11]	4	Case series

## Key differences between airplane (fixed-wing) and helicopter (rotor-wing) transport

- Acceleration: Airplanes provide smoother acceleration and deceleration compared to helicopters, minimizing sudden gravitational changes [25]. Such changes can cause fluctuations in ICP or cerebral perfusion, especially in those with the presence of intracranial air [9].
- Atmospheric Pressure: The pressurized cabin environment simulates an altitude of about 8,000 ft [25], resulting in a reduced partial pressure of oxygen. This can exacerbate hypoxia in patients with impaired respiratory or circulatory function and increasing intracranial pressure (ICP), risk of pneumocephalus, and gas embolism, for example in those with preexisting pulmonary pathology [42].
- Vibration and Noise: Vibration-induced stress and noise during helicopter transport, contribute to transient ICP fluctuations. Fixed-wing aircraft reduce these effects due to lower noise and vibratory forces reducing stress-induced increases in ICP and sympathetic stimulation [24, 31].
- Transport Duration: Fixed-wing aircraft typically cover longer distances, requiring prolonged patient monitoring and increased attention to ICP and oxygenation management [25].
- Temperature and Humidity: Airplane cabins typically maintain lower humidity levels (< 20%) [5], exacerbating dehydration, thickened secretions, impaired ciliary function, and thromboembolic events.
- Medical Team Accessibility: Fixed-wing aircraft offer greater space and accessibility for medical teams to perform patient interventions compared to rotor-wing aircraft.

The key differences listed mainly refer to the impact on cranial pathologies these factors are also likely to apply to patients with spinal pathologies. For example, trauma patients may have combined cranial and spinal pathologies, spinal fracture patients will require positioning to avoid acceleration stresses and hypoxia may cause exacerbation of spinal injuries.

## Notable case highlight

1 of the 106 reported patients deteriorated on route—Armitage J.M. et al., 1990 [4]. Here, “*Spontaneous respiration was adequate for 15 days, the patients then developed respiratory difficulties and a tracheostomy was performed. The next day the patient was flown to England breathing spontaneously, on arrival to London he was extremely dyspnoeic and had a respiratory arrest. He was ventilated and transferred to our unit. He required tracheal suction and physiotherapy to remove copious tenacious mucous plugs from his airways and required ventilation for 7 days*”. This highlights key points as normally in health inhaled air is warmed and humidified by the nose and oropharynx however this is bypassed with tracheostomy leading to prolonged inhalation of dry air. Dry air inspiration for an extended period causes the thickening of bronchial secretions and impaired ciliary function, thickened secretions may lead to obstruction and pulmonary collapse if not cleared. This may often be the case in patients with cervical and high thoracic spinal lesions as the abdominal and intercostal muscles are paralysed and the ability to cough and clear secretions becomes impaired. Additionally, the minimum barometric pressure in commercial aeroplanes is 75 kPa so the arterial oxygen pressure in healthy people is ~ 7.3kPa [4]. Therefore, patients already in a state of hypoxia may experience further deterioration e.g. bradycardia, during a flight if they are not given added oxygen. Incidence of bradycardia is reduced by preoxygenation before suction and skilful use of suction catheter and if appreciable bradycardia results IV atropine should be given. If patient is prone to serious bradycardia, they may be treated with oral adrenal agonist, e.g. orciprenaline, pre-flight [4].

### Summary of findings

The most salient points we extracted from the studies, which are especially important in patients with high spinal cord injuries, were:

- Lung function should stable before transfer.
- Humidification of inspired air should be adequate before and during transport (particularly for patients with tracheostomy)
- Supplemental oxygen available.
- Patients accompanied by someone who can clear secretions.
- IV Atropine available.

In this series all patients transferred immediately (< 36 h) or after 7/10 days. Not transferring patients between day 2–7 avoids the highest risk period for complications of the spinal cord injury and deterioration from that including spinal cord swelling, spinal cord shock. Some of the older papers mention traction of the patient during transfer which optimally should be avoided [11, 43].

## Discussion

This scoping review provides a synthesis of existing evidence regarding the unique physiological stressors encountered by spinal pathology patients during fixed-wing air transport. Here, we provide further contextualisation, by aggregating our combined experience to highlight key factors not addressed in the literature.

## Anecdotal evidence from experts

Our combined expertise is formed from an expert panel of consultant neurosurgeons, aviation lecturers, and spinal injuries clinical nurse specialist with vast experience of transferring acute spinally injured patients by fixed wing air transport, who has personally managed over 100 spinal pathology patients at altitudes of 30,000ft. This has enabled us to highlight nine key points not previously alluded to in the literature search:

1. The importance of keeping pressure points healthy in SCI (spinal cord injury) patients. “*Patients with SCI are at a high risk of pressure injuries (PI) due to their impaired mobility and lack of sensation to alert them to danger, this leads to pressure against points of tissue for extended periods of time causing impaired blood circulation and as such hypoxia and resultant PI*” [10]. Regarding air travel and pressure injuries, the “*US Air Force reports pressure injury rates of 4.9% between 2009–2014 in the transport of critically ill patients, with a high-risk subset of unstable thoracolumbar vertebral injury*” [13].
2. For patients which are ventilated, the cuff on the endotracheal tube (ETT) needs to be deflated by ~ 1/3. This recommendation is because at higher altitudes (approximately 30,000 ft), the expansion of gases following Boyle's Law, is proportional to pressure changes. Barometric pressure decreases by approximately 30 mbar/1000ft and therefore if not accounted for the ETT will inflate with increasing altitude. Endotracheal tube cuffs must be partially deflated to prevent excessive pressure on tracheal walls, which could result in ischemia and tracheal necrosis [36]. Conversely, this method may lead to leakage around the cuff during descent due to ETT deflation with reduced altitude which predisposes to aspiration pneumonia. To avoid these changes, an alternative method is to fill the cuff with normal saline, maintaining a steady pressure throughout the flight and as such is often a more stable option [2].
3. In patients with bowel and bladder sphincter problems it is vital to make sure patients are not constipated or obstructed and have a urinary catheter inserted. Not only is incontinence in an aircraft difficult to manage practically, but as gas expands at altitude (Boyle’s Law), the patient can develop diaphragm splitting if there is excessive gas, placing them at higher risk of respiratory failure [14]. Notably, the literature does not cover GI side effects of travel at altitude, which our experience leads us to believe is crucial for safe transport.
4. In acutely spinally unwell patients at altitude, it is extremely important to consider (venous thrombosis embolism) VTE prophylaxis so patients must have graduated compression stockings and prophylactic low molecular weight heparin. Spinal trauma/pathology carries a significant risk for VTE—“*patients with acute spinal cord injury (SCI) have the highest risk of VTE among hospitalised patients. The incidence of total deep vein thrombosis ranges from 50 to 100% in untreated patients*” [46]. This increased risk of VTE is exacerbated by air travel due to the physiological changes we alluded to earlier: hypoxia, dehydration, immobility during travel, and colder temperatures. Moreover, another paper concluded that “Air travel is a risk factor for VTE and there is a dose effect starting at 4 h” [38].
5. Diaphragm fatigue is underestimated as a case of respiratory failure within the first week of injury. As the respiratory workload increases at higher altitudes, patients are more likely to drift into type 2 respiratory failure [7].
6. Patients with high thoracic and cervical SCI can become poikilothermic due to loss of sympathetic control over thermoregulation [27]. Rapid environmental transitions during transport (ambulance → outdoors → aircraft) can lead to hypo- or hyperthermia, both of which significantly impact outcomes [26]. An example case from experience—a young male with an acute traumatic cervical injury from an RTC was transferred from the National Spinal Injuries Centre at Stoke Mandeville Hospital via HEMS from the scene of the accident. They were transferred in a vacuum mattress (which traps heat). Total transfer time was 40 min, and the patient had a core temperature of 40.5 degrees centigrade.
7. Control of blood pressure in SCI patients during transfer. Spinal shock leads to neurogenic hypotension due to vasodilation and bradycardia, particularly during the early spinal shock phase. Volume expansion is often ineffective, and vasopressors are typically required [41]. Excess fluids should be used very judiciously as they risk pulmonary oedema due to decreased sympathetic tone and venous return, which can precipitate respiratory failure.
8. Secretion retention is a major cause of morbidity in cervical SCI [22]. During transport, patients can quickly develop hypoxia and atelectasis if secretions are not cleared [29]. The transfer clinicians should be competent in manual assisted cough (MAC) techniques.
9. Silent aspiration (without cough or overt symptoms) is a complication of cervical SCI due to impaired pharyngeal function, reduced sensation, and poor coordination of swallow and breathing. It is under-recognised during transfers and can cause delayed-onset pneumonia or respiratory arrest [53].

However, as these findings have not been validated by further studies, they are Level 5, Grade D recommendations.

## Stressors of flight

Below, we interpret key findings, integrating clinical, engineering, and physiological perspectives, to analyze their implications through the lens of Boyle’s Law and gas dynamics, and address the unique challenges of emergency evacuation for acute spinal patients.

The interplay between fixed-wing aviation environments and spinal injury pathophysiology reveals multifaceted risks. Reduced cabin pressure (simulating ~ 8,000 ft altitude) [25] and prolonged flight durations amplify physiological stressors, including hypoxia, dehydration, and mechanical instability. Clinical data and military case studies emphasize the vulnerability of spinal patients to these conditions, particularly those with compromised respiratory function or unstable fractures. For instance, cervical spinal injuries impair cough reflexes and mucociliary clearance, exacerbating respiratory complications in low humidity cabins [5]. Similarly, vibration transmission, though reduced compared to rotor-wing transport, may destabilize spinal segments unless mitigated by advanced immobilization systems [31].

Engineering innovations, such as real-time vibration monitoring and adaptive pressurization protocols, hold promise for minimizing these risks. However, the current reliance on low-grade evidence (e.g., case reports, animal studies) limits the generalizability of recommendations [55]. This underscores the need for human trials in simulated flight environments to quantify outcomes like intracranial pressure (ICP) fluctuations and spinal perfusion under hypobaric conditions.

## The implications of Boyles Law on air transportation of SCI patients

Boyle’s Law, which dictates that gas volume inversely correlates with pressure (P_1_V_1_ = P_2_V_2_) when temperature remains constant, plays a crucial role in assessing altitude-related risks. At 8,000 ft, cabin pressure reductions cause enclosed gases to expand by approximately 25%, posing critical threats to spinal patients [57]. Neurological compromise may occur when post-surgical pneumocephalus or epidural air pockets displace neural structures, potentially elevating intracranial pressure and exacerbating spinal cord compression. In the bowel and respiratory systems, intestinal gas expansion can impair diaphragmatic function, while tracheal cuff overinflation, which is caused by expanding gas, risks tracheal ischemia. Although partial deflation of endotracheal tube cuffs prior to ascent aligns with Boyle’s Law, this practice is inconsistently applied in spinal-specific protocols. Emergency interventions such as in-flight procedures (e.g., needle thoracostomy for pneumothorax) require anticipatory adjustments to accommodate gas volume changes, highlighting the importance of altitude-aware clinical training.

## The implications of Dalton’s Law on air transportation of SCI patients

Daltons Law states that the total pressure of a given mixture of gases is equal to the sum of the partial pressures of each gas present (P_total_ = P_1_ + P_2_ + …) [2]. Therefore, as partial pressures decrease with increasing altitude the total atmospheric pressure decreases, which becomes significant in the air transport of spinal patients due to the decrease in the partial pressure of oxygen (PO_2_). At the standard-maintained cabin pressure of ~ 8,000ft, PO_2_ falls to ~ 118 mmHg, from ~ 159 mmHg at sea level [2]. This correlates to a decrease of partial alveolar pressure (PAO_2_) to 53–64 mmHg (from normal 99.7 mmHg) producing SpO2 85–91% [2]. This causes an increased risk of hypoxia especially in patients where baseline PaO_2_ values are already reduced. Spinal patients are often at an innate risk of hypoxia with respiratory complications being the most common cause of morbidity in SCI [7], complications such as pneumonia, atelectasis, inability to maintain airway patency due to impaired secretion clearance, reduced respiratory muscle strength/fatigue and autonomic system dysfunction. Patients with SCI will also be at a greater risk of deterioration due to hypoxia potentially resulting in further neuronal ischaemia/necrosis and impaired tissue repair. This highlights the importance of maintaining the patient’s airway via secretion clearance and the appropriate availability of oxygen supplementation or ventilation when required.

## The implications of Henry’s Law on air transportation of SCI patients

Henry’s Law dictates that the amount of a gas that is dissolved into a liquid is directly proportional to the partial pressure exerted by that gas over the solution (C = k * P) [2]. Therefore, as altitude increases and partial pressures decrease gas bubbles may form in tissues where the gas was previously dissolved, this can cause aerospace decompression sickness (ADI) [16]. These gas bubbles, which are usually nitrogen gas, can form specifically in the spinal cord, a form of type II decompression illness (which involves gas bubble formation in the CNS), activating inflammatory pathways, causing cord compression and contributing to axonal degeneration and demyelination [16]. This can lead to a range of symptoms such as paraesthesia, paralysis and urinary and faecal incontinence [16]. It is important to note that ADI usually only occurs in barometric pressures equivalent to that of 25,000ft or in individuals that have recently been diving, therefore for standard commercial or medical flights in which the cabin pressure is maintained at the equivalent of 8,000ft ADI is unlikely to occur unless there are failings of the pressurization systems. Additionally, regarding Henry’s law there is a risk to patients with underling pulmonary pathologies of developing air embolisms, pathologies such as cystic adenomatoid malformation, bronchogenic cysts or pulmonary bullae [42]. This may cause a sudden presentation of symptoms similar to that of a cerebrovascular accident, myocardial infarction or acute hypoxemic respiratory failure if pulmonary circulation is implicated [42]. Due to the diverse range of symptoms that both ADI and air embolism may cause they are important for clinicians to be aware of and consider in the event of spinal patient deterioration when transferring via fixed wing air transport.

## Broader literature on the aeromedical environment and acutely unwell patients

Many physiological stressors encountered in spinal pathology transfers mirror those seen in general ICU or trauma aeromedical retrievals but are often amplified by the vulnerability of the spinal cord. Guidelines from services such as the Royal Flying Doctor Service (RFDS) of Australia and London’s Helicopter Emergency Medical Service (London HEMS) emphasise similar priorities: maintaining oxygenation, preventing hypothermia, and minimising vibration and acceleration forces. RFDS routinely advocates in-flight humidification, early thromboembolism deterrent stockings, and careful bladder management in immobilised patients [14, 48] measures echoed in our spinal-specific recommendations.

Across critical care air transport, respiratory deterioration is the leading in-flight complication, often due to secretion retention, mucosal drying, or atelectasis [4, 25]. High thoracic and cervical injuries further predispose patients to respiratory failure from diaphragm fatigue [59]. Pre-flight optimisation, e.g. humidified oxygen and secretion clearance is essential to prevent crises, particularly in those with pre-existing hypoxia [14]. Other reported complications include new arrhythmias and unplanned sedation, with higher risk linked to vasopressor use and longer flight distances [50].

Effective immobilisation, such as vacuum mattresses or modular restraint systems, mitigates vibrational and gravitational forces; military data support their use in spinal fracture evacuation [32], though uptake in civilian systems is inconsistent. Prolonged immobility and hypobaric hypoxia synergistically raise venous thromboembolism (VTE) risk, particularly on flights > 4 hours [38]. Routine pharmacological and mechanical prophylaxis is recommended [28], though altitude-adjusted LMWH dosing protocols remain unvalidated.

Timing also matters: immediate transfer (< 36 h) benefits surgically accessible lesions, whereas delaying transport during the 2–7 day peak oedema phase can reduce secondary injury [12]. Medical repatriation of spinal cord injury patients, often over long distances after variable initial care, shares many of these risks despite occurring in the subacute phase [18].

Aligning spinal-specific recommendations with established trauma and critical care aeromedical standards strengthens a unified safety framework for fixed-wing transfers.

**Fig. 2:**
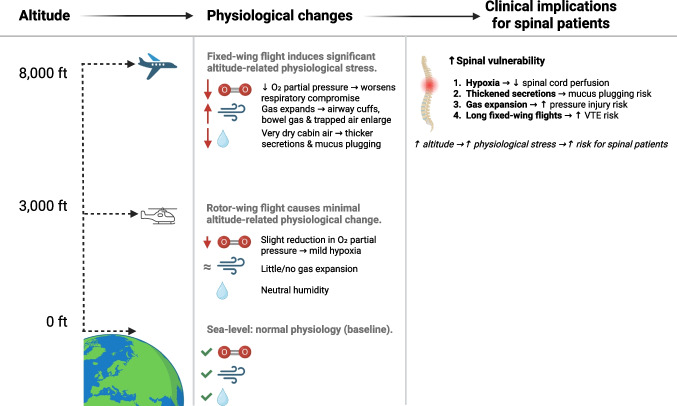
Visual representation of the physiological changes that occur at fixed-wing vs rotor-wing transport. Footnote: some specialised fixed-wing aeromedical aircraft pressurise the patient compartment, although cabin pressure is often set to an equivalent altitude lower than cruising altitude [34]

Key physiological concerns: (Fig. 2).

- Reduced atmospheric pressure → hypoxia risk
- Low cabin humidity → dehydration & respiratory complications
- Vibration/acceleration → potential ICP increase & spinal instability

## Summary of clinical recommendations

- Optimise respiratory status; ensure humidification and oxygen as needed, with trained staff for secretion clearance.
- Transfer within 36 h or after 7–10 days post-injury to avoid peak cord swelling.
- Prevent pressure injuries through monitoring and bowel/bladder care.
- Manage airway cuff pressures to prevent tracheal injury.
- Use low molecular weight heparin and graduated compression stockings to reduce venous thromboembolism risk, especially on long flights.

## Limitations and strengths

A significant finding from this review is the evident scarcity of rigorous, high-grade evidence on fixed-wing transport of spinal pathology patients, highlighting a critical gap in clinical guidelines. Most of the available data are derived from case reports and military cohort studies, which limits their generalizability and underscores the need for more systematic observational studies or clinical trials. However, we ultimately managed to identify 106 patients with spinal injury including 1 case of spinal TB. Other acute spinal cord pathologies, including spinal cord stroke or acute malignant spinal cord compression, were not identified from this literature search though many of the same conditions will be relevant.

The strengths of this review include the comprehensive literature search and expert contextualization, enhancing the practical relevance and applicability of the findings, especially as to our knowledge, this is the first review investigating this topic. However, limitations arise from the descriptive and heterogeneous nature of the existing studies, resulting in their low-level 4 and low-C grade classification by the Oxford Centre for Evidence Based Medicine. Clearly, as the experiences outlined by our experts are anecdotal, they need validation by further studies to form explicit conclusions and are only level 5, grade-D recommendations.

## Future directions

To improve the safety and outcomes of spinal pathology patients during fixed-wing transport, targeted technological and policy innovations are urgently needed.

1. Clinical-Aviation Integration:
  Aviation engineering must be harnessed to meet the physiological demands of medically vulnerable patients. One high-impact innovation is the development of *smart cabin systems*, integrated sensor arrays capable of real-time monitoring of intracranial pressure, tracheal cuff pressure, and end-tidal CO₂ [1, 17, 33, 40]. Linking this data to cockpit and medical crew displays would enable dynamic, in-flight decision-making to pre-empt complications.
2. Altitude Protocol Reform:
  Cabin pressurization protocols should be re-evaluated, with strong consideration for reducing cabin altitude to ≤6,000 ft during medical evacuation of patients with gas-sensitive pathologies [37]. Although this may marginally reduce aircraft fuel efficiency, the clinical benefit, especially for patients at risk of gas expansion, hypoxia, or ICP spikes warrants serious investigation.
3. Standardisation of Care Protocols:
  Immediate efforts should focus on developing evidence-based, internationally endorsed protocols for key aspects of care: spinal immobilisation, thromboembolism prophylaxis [15], pressure injury prevention, and airway management at altitude. Existing military data on vacuum mattress use and transfer timing should be used to inform civilian adaptations.
4. Structural Collaboration:
  Multinational task forces comprising neurosurgeons, aerospace engineers, critical care specialists, and regulators, should be formally convened to translate current insights into enforceable operational standards [20] and identify evolving gaps in the literature, as recommended by Arksey and O’Malley [3]. These bodies must prioritise cost-effective adaptations for low- and middle-income countries, such as modular immobilisation kits, portable suction devices, and oxygen-humidification units.
5. International Data Collection and Quality Registries:
  An international, standardised quality registry for aeromedical transport of spinal pathology patients would enable prospective data collection, facilitate meaningful comparison across transport modalities, and support the development of evidence-based clinical guidelines. Establishing such a registry represents an important future research priority to address current evidence gaps and improve patient safety.

The unique needs of spinal trauma patients in air transport environments cannot be met by siloed innovation. Coordinated, interdisciplinary solutions are essential to transform isolated knowledge into systematic, safe practice. Future clinical trials in simulated cabin environments, including hypobaric chambers and aviation medical simulators, are urgently needed [19] to validate proposed physiologic safeguards and optimise transfer protocols.

## Conclusion

Specific, evidence-based guidelines are urgently needed to address the unique physiological challenges faced by spinal pathology patients during fixed-wing air transport. Although existing literature highlights key priorities e.g. respiratory optimisation, timing of transfer, and prevention of pressure injuries and venous thromboembolism, current insights are fragmented and grounded largely in low-level evidence. This review links fundamental physiological principles, including Boyle’s, Dalton’s, and Henry’s laws, with their real-world implications at altitude, combining expert experience with broader aeromedical data. Fixed-wing evacuation poses distinct risks compared with rotor-wing or ground transport, demanding customised strategies that integrate medical and engineering expertise. Progress will depend on translational research, robust observational studies, and structured cross-disciplinary collaboration to develop standardised protocols and improve the safety and effectiveness of spinal patient aeromedical evacuation worldwide.

## Supplementary Information

Below is the link to the electronic supplementary material.

ESM 1 Supplementary Material 1 (DOCX 16.4 KB)

ESM 2 Supplementary Material 2 (PDF 303 KB)

## Data Availability

All data available in this publication and its supplementary files.
