# Supplementary material for: Fixed-wing air transport of patients with spinal pathologies: a scoping review of current evidence and future research priorities
Source: Acta Neurochir (Wien). 2026 Apr 2;168(1):94. doi: 10.1007/s00701-026-06852-0 (PMC13053554; doi:10.1007/s00701-026-06852-0)
Supplement: Supplementary file 1 — Supplementary Material 1 (DOCX 16.4 KB) [file 701_2026_6852_MOESM1_ESM.docx]

**Appendix 1: Search strategies**

**OVID Search strategy:**

AMED (Allied and Complementary Medicine) <1985 to August 2024>

Books@Ovid <September 18, 2024>

Embase Classic+Embase <1947 to 2024 September 20>

Emcare 1995 to present

GEOBASE <1980 to July 2024>

GeoRef <1666 to September Week 02 2024>

GeoRef's InProcess <July 2024>

Global Health <1910 to 2024 Week 38>

Health and Psychosocial Instruments <1985 to July 2024>

ICONDA <1976 to August 2024>

International Pharmaceutical Abstracts <1970 to September 2024>

Journals

Maternity & Infant Care Database (MIDIRS) <1971 to September 10, 2024>

Ovid MEDLINE(R) ALL <1946 to September 19, 2024>

APA PsycArticles Full Text

APA PsycBooks <1806 to August 2024>

APA PsycExtra <1908 to September 09, 2024>

APA PsycInfo <1806 to September 2024 Week 3>

APA PsycTests <1910 to September 2024>

APA PsycTherapy <August 2024>

Social Policy and Practice <202408>

Transplant Library <August 28, 2024>

HMIC Health Management Information Consortium <1979 to July 2024>

1          Air ambulance/           9281

2          Medical airlift.ti,ab.    11

3          (Medical adj2 (air or aerial or air medical or aeromedical or aeronautical or helicopter or fixed wing) adj2 (evacuation$ or transfer$ or transport$ or transportation$ or travel$ or flight$ or ambulance or airlift$)).ti,ab.   2046

4          ((Air or aerial or air medical or aeromedical or aeronautical or helicopter or fixed wing) adj2 (evacuation$ or transfer$ or transport$ or transportation$ or travel$ or flight$ or ambulance or airlift$)).ti,ab. 32230

5          ((Air or aerial or air medical or aeromedical or aeronautical or helicopter or 'fixed wing') adj2 patient$ adj2 (evacuation$ or transfer$ or transport$ or transportation$ or travel$ or flight$ or ambulance or airlift$)).ti,ab.   830

6          ((National or international) adj2 (air or aerial or air medical or aeromedical or aeronautical or helicopter or 'fixed wing') adj2 (evacuation$ or transfer$ or transport$ or transportation$ or travel$ or flight$ or ambulance or airlift$)).ti,ab.   833

7          1 or 2 or 3 or 4 or 5 or 6         37530

8          Spine surgery/ 50497

9          (Patient$ adj2 (with or undergoing or after) adj2 (spine or spinal or 'post-spinal' or 'post-operative' or postoperative) adj2 (surgical or surgery or operation)).ti,ab.    9020

10        ((Spine or spinal or 'post-spinal') adj2 (surgery or surgical or operation) adj2 patient$).ti,ab.          9657

11        (Patient$ adj2 (spine or spinal or post-spinal or post-operative or postoperative) adj2 (surgical or surgery or operation)).ti,ab.    10828

12        (Postoperative adj2 (spine or spinal) adj2 (surgical or surgery or operation)).ti,ab.  355

13        (Patient$ adj2 (spine or spinal or 'post-spinal' or 'post-operative' or postoperative) adj2 (surgical or surgery or operation)).ti,ab. 10828

14        8 or 9 or 10 or 11 or 12 or 13 62751

15        Spinal cord injury/      141787

16        ((cervical or thoracic or lumbar or sacral or saccral) adj2 (injury or fracture or tumour or tumor or infarct or hematoma or haematoma)).ti,ab.      37493

17        ('Spinal degenerative condition' or 'degenerative disc disease' or 'spinal stenosis' or 'osteoarthritis of the spine' or 'facet joint syndrome' or spondylosis or Spondylolisthesis or 'Herniated Disc' or 'Bulging Disc' or 'Spinal Disc Desiccation' or 'Degenerative Scoliosis' or 'Ankylosing Spondylitis').ti,ab.       118256

18        ('traumatic injury' or 'traumatic injuries' or 'compression fracture$' or 'burst fracture$' or 'chance fracture$' or 'fracture dislocation' or 'complete spinal cord injury' or 'incomplete spinal cord injury' or 'cord injury' or whiplash or 'spinal contusion' or 'dislocation of vertebrae').ti,ab.            228316

19        ((spinal or spine) adj2 (oedema or edema or compression)).ti,ab.     26846

20        ('infectious disease$' or 'Spinal Osteomyelitis' or 'Spinal Tuberculosis' or 'Pott's Disease' or 'Potts disease' or 'Epidural Abscess' or Discitis).ti,ab. 424479

21        cauda equina/  12834

22        (neoplastic or cancer or 'spinal tumour$' or tumour$ or 'Metastatic spinal tumor$' or meningioma or schwannoma or astrocytoma or 'Multiple Myeloma').ti,ab.      7861004

23        ('vascular condition' or 'spinal cord infarct' or 'spinal cord infarction' or 'spinal AVM' or 'spinal Arteriovenous Malformation' or 'Spinal Hematoma' or 'spinal haematoma').ti,ab.        5343

24        (Syringomyelia or 'Chiari Malformation' or Myelopathy).ti,ab.        64752

25        15 or 16 or 17 or 18 or 19 or 20 or 21 or 22 or 23 or 24        8741027

26        14 or 25           8789838

27        7 and 26          1425

28        limit 27 to english       1337

29        animal/            13784622

30        28 not 29         1154

**Air Medical Journal Search strategy:**

1       neurosurgery*; NOT helicopter      23

**World Neurosurgery Search strategy:**

via ScienceDirect

1        World Neurosurgery     23611

2        'air medical transport' NOT 'helicopter'     61

**CNS Neurosurgery Search strategy:**

1        'air medical transport'; NOT helicopter      1016

2        ‘Articles’ content type filter      717

3        ‘Spine’ subject filter       7

**Journal of Neurosurgery Search strategy:**

1        Full text: airplane       74

2       OR Full text: aeroplane       74

3       OR Full text: fixed wing transport       86

4       OR Full text: aviation       146

5       AND Full text: spine/       61

6       NOT Full text: helicopter       48
